# Exploring Stem-Cell-Based Therapies for Retinal Regeneration

**DOI:** 10.3390/life14060668

**Published:** 2024-05-23

**Authors:** Madalina Radu, Daniel Constantin Brănișteanu, Ruxandra Angela Pirvulescu, Otilia Maria Dumitrescu, Mihai Alexandru Ionescu, Mihail Zemba

**Affiliations:** 1 Department of Ophthalmology, “Dr. Carol Davila” Central Military Emergency University Hospital, 010825 Bucharest, Romania; 2 Department of Ophthalmology, University of Medicine and Pharmacy “Grigore T. Popa”, 700115 Iasi, Romania; 3 Department of Ophthalmology, University of Medicine and Pharmacy “Carol Davila”, 050474 Bucharest, Romania; 4 Department of Ophthalmology, University Emergency Hospital, 050098 Bucharest, Romania

**Keywords:** stem cell, stem cell therapy, retinal degenerative diseases, age-related macular degeneration, retinitis pigmentosa, Stargardt’s disease, embryonic stem cells, induced pluripotent stem cell, mesenchymal stem cells

## Abstract

The escalating prevalence of retinal diseases—notably, age-related macular degeneration and hereditary retinal disorders—poses an intimidating challenge to ophthalmic medicine, often culminating in irreversible vision loss. Current treatments are limited and often fail to address the underlying loss of retinal cells. This paper explores the potential of stem-cell-based therapies as a promising avenue for retinal regeneration. We review the latest advancements in stem cell technology, focusing on embryonic stem cells (ESCs), pluripotent stem cells (PSCs), and mesenchymal stem cells (MSCs), and their ability to differentiate into retinal cell types. We discuss the challenges in stem cell transplantation, such as immune rejection, integration into the host retina, and functional recovery. Previous and ongoing clinical trials are examined to highlight the therapeutic efficacy and safety of these novel treatments. Additionally, we address the ethical considerations and regulatory frameworks governing stem cell research. Our analysis suggests that while stem-cell-based therapies offer a groundbreaking approach to treating retinal diseases, further research is needed to ensure long-term safety and to optimize therapeutic outcomes. This review summarizes the clinical evidence of stem cell therapy and current limitations in utilizing stem cells for retinal degeneration, such as age-related macular degeneration, retinitis pigmentosa, and Stargardt’s disease.

## 1. Introduction

Within the broad spectrum of human sensory capabilities, vision undoubtedly holds a paramount position. The clarity and acuity of vision are closely linked with an individual’s quality of life, influencing everything from daily tasks to broader social interactions. Many irreversible retinal diseases are marked by the progressive degeneration of retinal neural cells. Notably, age-related macular degeneration (AMD) persists as a dominant cause of severe visual impairment, especially in industrialized nations, emphasizing the pressing need for efficacious therapeutic strategies. While AMD needs significant attention due to its prevalence, other retinal degenerative disorders, such as retinitis pigmentosa and Stargardt’s disease, also inflict considerable visual deficits on affected individuals [1,2].

In the complex landscape of retinal degenerative diseases, the initiation and progression of these disorders involve a nuanced interplay between various cellular components of the retina. Notably, the retinal pigment epithelium (RPE) and photoreceptors constitute a critical partnership, essential for visual function. The RPE, a monolayered epithelium, plays a pivotal role in the survival, integrity, and functionality of photoreceptors by forming an intricate connection with their outer segments. However, the pathogenesis of retinal diseases often triggers a cascade of cellular degeneration, commencing with either the RPE or photoreceptors [3,4].

As these diseases advance, a broader spectrum of retinal components, including ganglion cells and the microvascular network comprising endothelial cells and pericytes, undergoes degeneration. This widespread cellular loss highlights the intricacies of retinal degenerative diseases and underscores the urgency for developing multifaceted therapeutic approaches. This review specifically focuses on macular degenerative diseases, including AMD, retinitis pigmentosa, and Stargardt’s disease, acknowledging the unique challenges and therapeutic targets they present [5].

The pursuit of effective treatments for retinal degenerative disorders has led to the exploration of stem cell replacement therapy as a promising avenue. Stem cell therapy aims to rejuvenate the retina by replacing deteriorated cells with healthy ones and/or rescuing retinal neurons from further degeneration, potentially restoring vision [6]. The initial forays into ocular stem cell therapy were directed towards the cornea, benefiting from its accessibility and simpler structure [7]. These efforts established the foundation for future investigations focused on the retina, which is a tissue that is more intricate in structure, has a rich blood supply, and comprises an extensive variety of different types of cells [7].

The field of stem cell therapy for the retina has witnessed significant advancements since those early days. The discovery of human embryonic stem cells (ESCs) [8] in the late 1990s marked a pivotal moment, offering unprecedented possibilities for regenerative medicine. Despite ethical and practical challenges, the subsequent development of induced pluripotent stem cells (iPSCs) [9] in 2006 provided a more versatile and ethically acceptable source of stem cells. These innovations have significantly advanced the application of stem cell therapies in addressing retinal degenerative diseases.

Today, the field of stem cell treatment for retinal disorders includes a range of strategies, from transplanting ESC-derived retinal pigment epithelium cells to utilizing autologous iPSCs for personalized medicine. Recent advances also emphasize allogenic stem cell approaches, which utilize donor-derived cells to provide ready-to-use, standardized treatments. These allogenic therapies offer advantages in scalability and consistency, but they require careful management of immune rejection risks through enhanced immunomodulatory techniques [10]. Clinical trials and research efforts are increasingly focused on optimizing delivery methods, enhancing cell integration and survival, and ensuring long-term safety and efficacy. As the field continues to advance, the integration of stem cell therapy into clinical practice emerges as a significant source of optimism for individuals with retinal degenerative disorders.

## 2. Materials and Methods

An extensive review of the existing literature was undertaken utilizing the PubMed digital database to locate relevant publications, complemented by a systematic search of clinical trials on ClinicalTrials.gov. The investigation focused on key terms such as “stem cell”, “stem cell therapy”, “retinal degenerative diseases”, “age-related macular degeneration”, “retinitis pigmentosa, “Stargardt’s disease”, “embryonic stem cells”, “induced pluripotent stem cell”, and “mesenchymal stem cells” to encompass a broad spectrum of research in this field. All pertinent articles published in English were included in this review. Additionally, articles in other languages were considered if they were accompanied by a detailed summary and an English abstract, ensuring a comprehensive understanding of global advancements. The references of all articles were meticulously examined to uncover further significant studies.

## 3. Stem Cell Sources

Significant progress has been achieved in stem cell research since the isolation of embryonic stem cells from a mouse embryo in 1981 and, subsequently, from a human embryo in 1998. The advent of human embryonic stem cells was a notable milestone, notwithstanding the ethical debates and financial limitations in place in 2001. The emergence of induced pluripotent stem cells in 2006, as pioneered by Japanese scientists, presented a very promising and morally more acceptable alternative that has comparable pluripotency and self-renewal attributes [9].

In addressing the challenges posed by the microenvironment in degenerative retinal diseases, it is vital to consider the inherent properties of the retina and the pathological changes these diseases induce [11,12]. The progression disrupts the delicate architecture essential for cellular survival and integration of transplanted cells. Current stem cell therapy trials are exploring strategies such as cell preconditioning, scaffold use for structural support, and co-delivery of trophic factors to modify the adverse microenvironment [13]. These approaches are essential in overcoming the obstacles that limit the efficacy of regenerative therapies in the retina.

The initiation of the first FDA-approved clinical study with human embryonic stem cells took place in 2009, with a specific emphasis on investigating spinal cord injuries. Subsequently, in 2010, the scope of stem cell research expanded to include the exploration of therapeutic interventions for retinal diseases [14]. The retinal pigment epithelium is pivotal in the pathogenesis of several degenerative retinal disorders, marking it as a significant target for regenerative therapies. Its unique properties, such as operational independence from neuronal synapses and the ability to function within the traditionally immune-privileged environment of the eye, underscore its therapeutic promise [15]. However, the notion of immune privilege is nuanced, particularly in disease states that may compromise this status, thus heightening immune activity within the RPE and subretinal space. This complexity does not diminish the RPE’s therapeutic potential but, rather, highlights the need for a sophisticated understanding of its interactions within the ocular immunological environment, especially under pathological conditions [16]. Among this, the exploration of various stem cell types, including human embryonic stem cells, induced pluripotent stem cells, and mesenchymal stem cells, continues to advance, offering hope for regenerative treatments that can address the multifaceted nature of retinal illnesses.

Within the sphere of regenerative medicine, the decision to use autologous (derived from the patient) versus allogeneic (derived from a donor) stem cells presents a nuanced set of advantages and challenges. Autologous stem cells minimize the risk of immune rejection and are considered safer for the patient. However, they might harbor inherent genetic anomalies that could potentially compromise the treatment’s effectiveness, a concern particularly pertinent to retinal disorders known for their genetic foundations [17]. In contrast, allogeneic stem cells, sourced from donors, bypass the issue of the patient’s genetic defects but carry the risk of immune incompatibility [18]. The advent of induced pluripotent stem cells offers a promising solution by creating patient-tailored stem cells with reduced immune reaction risk, although the reprogramming process raises concerns about possible genetic instabilities [19]. Therefore, the application of stem cells in therapies necessitates meticulous genetic assessment and profiling to ensure the cells’ safety and efficacy, striving to balance the therapeutic benefits against genetic risk factors.

### 3.1. Human Embryonic Stem Cells

Embryonic stem cells (ESCs) are a distinct category of pluripotent cells derived from the inner cell mass of blastocysts. The cells in question have a notable capability for autonomous regeneration and possess the potential to undergo differentiation into several cell lineages seen in mature organisms. This differentiation may occur in any of the three fundamental germ layers, namely, the endoderm, ectoderm, and mesoderm layers. After the blastocyst stage, which typically occurs about 4 to 5 days post-fertilization, the embryonic cells begin the process of differentiation. This intricate process results in the development of distinct cell types that comprise diverse organs, such as the heart and nerve cells. As a result, these cells can no longer be categorized as stem cells. Since their discovery in 1998, hESCs have emerged as a key resource in regenerative medicine, showing promise in their ability to repair and transform into various cell types [8].

Human embryonic stem-cell-based therapy for retinal diseases is being looked into because ESCs have the ability to repair and change into different types of cells. They have shown increased telomerase activity, indicating their potential for prolonged lifespan. Furthermore, it has been shown that these cells exhibit certain markers often associated with undifferentiated cells [8]. According to the theory of pluripotency, these cells can change into any type of cell found in an adult organism. This includes retinal pigment epithelial (RPE) cells when they receive the right signals [20].

The survival of human embryonic stem cell-derived retinal pigment epithelium (hESC-RPE) cells in the subretinal microenvironment is crucial for the therapeutic restoration of vision in patients with retinal degenerative diseases. A significant barrier to the long-term success of these cell therapies is the immunogenicity of transplanted cells, which can lead to the rejection and failure of the implant. Traditional approaches rely heavily on systemic immunosuppression, which poses risks of increased infection and malignancy. Recent advancements in gene editing technologies, particularly CRISPR-Cas9 [21,22], offer promising strategies to mitigate these risks by reducing the immunogenicity of hESC-RPE cells. Gene editing can be employed to modify specific gene sequences responsible for eliciting immune responses. For example, disrupting the beta-2 microglobulin (B2M) gene diminishes the expression of major histocompatibility complex (MHC) class I molecules, thus reducing the visibility of these cells to cytotoxic T cells [23]. Similarly, knockdown of the Class II Major Histocompatibility Complex Transactivator (CIITA) leads to decreased expression of MHC class II genes, essential for antigen presentation to helper T cells [24]. Such modifications have shown potential in preclinical models to enhance the survival of hESC-RPE cells without necessitating prolonged immunosuppression. However, the application of gene editing raises concerns regarding off-target effects and the genetic stability of edited cells. Ongoing research is thus directed at enhancing the precision of gene-editing techniques to ensure the safety and efficacy of these hypoimmunogenic cells in clinical settings [21]. The evolving regulatory landscape will also play a critical role in the clinical translation of these gene-edited cell therapies, ensuring they meet safety standards without compromising therapeutic benefits.

### 3.2. Induced Pluripotent Stem Cells

Induced pluripotent stem cells (iPSCs) are obtained from adult tissue and were first documented by Takahashi and Yamanaka in 2006 [9]. Derived from adult somatic cells, such as dermal fibroblasts, iPSCs undergo retroviral transduction to express genes associated with pluripotency, known as “reprogramming factors”. These factors, including transcription factors Oct 4, Sox 2, cMyc, and Klf4, enable the cells to acquire traits similar to embryonic stem cells [9].

Human-induced pluripotent stem cells (hiPSCs), derived from dermal fibroblasts, share key characteristics with hESCs, including appearance, gene expression, telomerase activity, cell division, and trilineage differentiation potential [25]. Due to the fact that induced pluripotent stem cells may be obtained from the same individual who will receive the iPSC-derived retinal pigment epithelium (iPSC-RPE) transplant, the transplanted tissue is not susceptible to immunological rejection. Consequently, this approach eliminates the need for systemic immunosuppression post-transplantation, thereby eliminating the associated risks. In vitro studies have shown that retinal pigment epithelial cells produced from induced pluripotent stem cells have the ability to partially suppress T cell proliferation and activation, perhaps mediated by the secretion of the soluble molecule transforming growth factor beta (TGFβ) [26].

Nevertheless, the use of induced pluripotent stem cells, while promising, is limited by a narrower range of experience and requires more extensive in vitro modification compared to human embryonic stem cells [27]. Additionally, the increased costs associated with collecting and manipulating autologous cells for transplantation could restrict large-scale production, thus impacting the economic viability of this method [27].

In considering cell-based therapies for retinal diseases, it is imperative to acknowledge the complexity of the retinal structure and the progressive nature of cellular degeneration, as discussed by Zhong et al. [28] and Hallam et al. [29]. These studies underline the inherent challenge in using a single type of cell for therapeutic purposes in conditions like AMD, where sequential death of retinal pigment epithelium (RPE) and photoreceptors occurs. The potential of hiPSCs and hESCs to differentiate into three-dimensional retinal structures that mimic the cellular organization of the human retina offers a promising avenue not only for studying disease mechanisms but also for developing regenerative therapies that address multiple affected cell types simultaneously [10,28]. This approach could potentially overcome the limitations of simpler, single-cell type transplants, aligning with the growing consensus that effective treatment for complex retinal diseases may necessitate the restoration of several layers of retinal cells to fully recuperate function [10].

Induced pluripotent stem cells present two notable advantages in comparison to ESCs. Firstly, iPSCs eliminate the necessity for ESCs, as they are generated through the reprogramming of mature adult somatic cells. Secondly, iPSCs offer the possibility of autologous production, allowing for the theoretical creation of individual-specific iPSC lines [25].

However, this technology is not without its drawbacks. Some of these are the relatively slow process of turning somatic cells into iPSCs, the chance of introducing genetic mutations through the transcription factors used in the process, and the chance that tumors will grow. This latter concern is particularly linked to the activation of oncogenes, both intentionally and unintentionally, by viruses utilized in the genomic modification of cells [30,31]. Recognizing these risks, recent advances have focused on developing non-viral methods for creating iPSCs and iPSC-RPE cells. Techniques such as mRNA transfection [32], which involves the introduction of synthetic mRNA sequences encoding reprogramming factors, offer a safer alternative by eliminating the risk of insertional mutagenesis associated with viral vectors. Additionally, the use of small chemical molecules that can induce reprogramming by altering cell signaling pathways, using the concept of transdifferentiation [33]—also known as direct lineage conversion—has also been explored. These methods not only reduce the potential for oncogene activation but also enhance the efficiency and safety of iPSC and iPSC-RPE generation, providing promising avenues for therapeutic applications without the drawbacks of viral integration [33]. In spite of the progress achieved with iPSCs, the sustained interest in employing embryonic stem cells for research persists, possibly attributable to the significant expense and extensive time required for the development of iPSCs.

### 3.3. Mesenchymal Stem Cells

Mesenchymal stem cells (MSCs), known for their multipotent capability, play a crucial role in regenerative medicine. Originating from stromal compartments of various tissues, including bone marrow, adipose tissue, and umbilical cord blood, MSCs exhibit remarkable plasticity, showing a unique ability to differentiate into diverse cell lineages, including osteoblasts, chondrocytes, and adipocytes. Recent advancements highlight their potential to differentiate into retinal cells, opening new avenues for treating degenerative retinal diseases [34].

The progressive loss of photoreceptors and retinal pigment epithelium cells that characterizes retinal degenerative diseases, such as AMD and RP, leads to irreversible vision impairment. Traditional therapeutic approaches have been predominantly palliative, with a limited capacity for reversing the degenerative process. The therapeutic efficacy of MSCs in retinal regeneration is primarily attributed to their secretion of a diverse array of trophic factors and cytokines, which provide neuroprotective effects and modulate the local retinal environment to support regeneration [35]. Moreover, MSCs are reported to possess anti-apoptotic properties [36] and can integrate into damaged retinal layers, potentially rejuvenating their structure and function. Notably, MSCs also exhibit immunomodulatory properties that may mitigate inflammation [37], a common pathological feature in degenerative retinal diseases. This dual functionality of MSCs—regenerative and immunomodulatory—enhances their potential as a transformative treatment for conditions that have previously been difficult to manage effectively. However, the efficacy of MSCs in treating macular degenerative diseases is significantly limited by the disease’s stage at the time of treatment. For example, in cases of advanced macular geographic atrophy, extensive loss of retinal cells may prevent significant recovery, marking a primary limitation of MSC therapy. Furthermore, treatment outcomes with MSCs can vary widely; while some patients may see slight improvements in vision, others may not experience any benefit, underscoring the inconsistent results in late-stage retinal degeneration [36]. This variability underlines the urgent need for further research to refine MSC delivery techniques and determine the optimal timing for intervention, aiming to improve therapeutic outcomes [35].

Bone marrow stromal cells (BMSCs) are found in the bone marrow and represent the highest proportion of adult stem cells. Two distinct types of BMSCs have been identified, mesenchymal stem cells and hematopoietic stem cells, with the latter also known as CD34+ cells. These cells are characterized by their multipotency, which, while more restricted than that of pluripotent stem cells, enables them to differentiate into various cell types [38,39]. Additionally, they exhibit paracrine trophic effects through the secretion of neurotrophic factors or anti-inflammatory modulators. MSCs, constituting less than 0.1% of bone marrow cells, can be efficiently expanded in vitro and are also present in other tissues, including teeth and the liver [34].

BMSCs offer several advantages. They possess the innate ability to migrate towards sites of injury and have the capacity for transdifferentiation, meaning they can adapt to differentiate into cells of different organs under specific environmental conditions [40]. In the context of retinal damage, such as that affecting RPE cells, they respond to chemo-attractive cytokines/chemokines released by the injured tissue, facilitating their migration to the site of injury. Once present, they have the potential to differentiate into retinal cells, including RPE cells and photoreceptors, aiding in tissue repair. Furthermore, BMSCs can produce neurotrophic factors that support cell survival and exert anti-inflammatory effects. An additional benefit of CD34+ cells is their ease of extraction from patients, coupled with minimal manipulation requirements, making them suitable for autologous transplantation procedures [38,40].

The heterogeneity of mesenchymal stromal cells, influenced by their tissue source, presents significant challenges in their clinical application and manufacturing consistency. MSCs derived from bone marrow, adipose tissue, and umbilical cord blood, for instance, show distinct properties that affect their therapeutic potential [36]. Different sources of MSCs exhibit unique profiles impacting their therapeutic applications [36]. Given these differences, the manufacturing process, particularly under current good manufacturing practices (cGMP), must adapt to maintain the unique characteristics of MSCs from each source [41]. This involves stringent control over the entire manufacturing process, from cell isolation through to expansion and storage, to ensure product safety, potency, and efficacy.

The integration of automated and closed-system bioreactors into MSC manufacturing is transforming production by standardizing procedures and significantly enhancing consistency across different production runs. Advanced automation technologies enable precise control of culture conditions—such as pH, temperature, and nutrient supply—enhancing cell quality and yield [41,42]. These systems also facilitate scalable production platforms that can accommodate the high throughput demands of clinical application, ensuring that consistent MSC products are manufactured across different batches. Such developments not only streamline the manufacturing process but also minimize variations introduced by manual handling, thereby enhancing the consistency of MSC batches irrespective of their source.

## 4. Clinical Studies

Clinical trials of stem cell therapy in degenerative macular diseases represent a cutting-edge frontier in ophthalmological research, focusing on retinal regeneration. Stem cells have the unique ability to grow new cells. This means that these trials could lead to new treatments for conditions like age-related macular degeneration and hereditary retinal dystrophies [43,44].

The core principle of these trials is the transplantation of stem cells into the retinal space, aiming to replace or repair the damaged retinal pigment epithelium and photoreceptors [6]. Various stem cell types—including embryonic stem cells, induced pluripotent stem cells, and adult stem cells—have been explored, each with their advantages and challenges [6].

Currently, the majority of conducted human clinical studies (Table 1) fall into the initial phase, focusing on safety assessment and lacking the statistical power necessary to discern functional outcomes. These studies have consistently employed the eye with poorer vision as the subject, using the other eye as a comparative but less-than-ideal control [45]. In the context of retinal pigment epithelium cells, their distinct pigmentation facilitates identification through ophthalmoscopy. Nonetheless, similar pigmentation is also observed in macrophages that are involved in the engulfment of deteriorating RPE cells [44].

It has been established through preclinical research that the detection of viable transplanted RPE cells is most reliably accomplished using immunohistochemistry [46]. As a result, in addition to documenting new pigmentation zones, there has been an increased reliance on diverse imaging modalities. Optical coherence tomography (OCT) has proven effective in revealing structural alterations within the subretinal space and the photoreceptor outer segments [47]. Fundus autofluorescence depends on lipofuscin, which is a byproduct of normal retinal processes and is missing in atrophied areas. This makes it hard to figure out what is going on after a transplant [47]. Various forms of electroretinogram (ERG), including multifocal and full-field, have been deployed to assess retinal function. Techniques such as visual field testing and microperimetry are instrumental in mapping scotomas resulting from atrophy. While tests for visual acuity and reading speed are feasible, their utility is reduced in studies focusing on patients with severely compromised baseline vision. There is a growing need to refine visual function testing methods, particularly for patients with low vision, to evaluate the efficacy of these treatments more effectively in cases of advanced-stage diseases [48].

### 4.1. Clinical Trials Using hESCs

Subsequent to the encouraging outcomes from preclinical investigations, the United States Food and Drug Administration sanctioned the commencement of Phase I/II clinical trials in 2010, focusing on stem cell therapies for retinal pathologies in human subjects. These trials employed retinal pigment epithelium cells derived from human embryonic stem cells. In 2012, Schwartz and colleagues (NCT01345006; NCT01344993) disseminated the inaugural findings of this pivotal study [49,50]. The procedure entailed the administration of hESC-RPE cells into the subretinal space, specifically targeting a pericentral region, accomplished via pars plana vitrectomy. This intervention was applied to individuals diagnosed with age-related macular degeneration and Stargardt’s disease (SD). The initial report, covering a follow-up period of four months, highlighted one patient with AMD and another with SD. Notably, in this duration, neither patient exhibited adverse effects such as tumorigenic proliferation, the development of ectopic tissues, nor signs of graft rejection, thereby indicating a preliminary safety profile of the procedure [49]. Subsequently, Schwartz provided an elucidation of the outcomes following a 22-month follow-up period involving 18 patients, comprising nine individuals with AMD and an equal number with SD. During this period, an improvement in the Best Corrected Visual Acuity was observed in ten cases, whereas it remained stable in seven instances.

However, there was a notable deterioration exceeding 10 letters in visual acuity in one case. In approximately 72% of the instances, there was an observable increase in pigmentation at the periphery of macular atrophy, aligning with the locations of the retinal pigment epithelium transplants. Adverse effects recorded in the course of the study included the development of endophthalmitis in one patient, cataract formation in four cases, and complications associated with the immunosuppressive treatment [50].

Participants enrolled in the study necessitated immunosuppressive therapy, entailing a regimen of low-dose tacrolimus, with targeted blood concentrations between 3 and 7 ng/mL, and mycophenolate mofetil, administered orally in doses varying from 0.25 to 2.00 g daily. This immunosuppressive protocol was initiated a week preceding the surgical intervention and sustained for a duration of 12 weeks post-therapy [50]. The rationale for this approach stemmed from the heterologous origin of the transplanted cells. Notwithstanding the implementation of immunosuppression, the patients demonstrated a notable enhancement in visual acuity.

Concurrently, Song et al. [51] (NCT01674829) conducted a one-year post-transplantation follow-up of four patients, comprising two with dry age-related macular degeneration and two with Stargardt’s disease, who underwent human embryonic stem cell-derived retinal pigment epithelium transplantation. Their observations revealed an absence of adverse proliferation or tumorigenicity. Echoing the findings of Schwartz et al. [50], this study noted an enhancement in visual acuity in three of the patients, while one patient exhibited stable vision.

Mehat et al. [52] (NCT01469832) conducted a comprehensive investigation to assess the safety of human embryonic stem cell-derived retinal pigment epithelium in the context of Stargardt’s Macular Dystrophy. This study encompassed 12 patients, each of whom received subretinal injections of hESC-RPE cells, with the cell count ranging from 50,000 to 200,000. Accompanying the procedure, all patients were administered a combination of Tacrolimus and Mycophenolate Mofetil (MMF) as immunosuppressants, with no reported complications stemming from their use [52]. Surgical challenges were noted in four patients, including instances of retinal dialysis, subretinal hemorrhage, and vitreous hemorrhage. Notably, there were no indications of immune rejection or undesirable proliferation of the transplanted RPE cells. Observation of subretinal pigmentation was uniform across all study participants, and optical coherence tomography imaging revealed a hyper-reflective layer congruent with the presence of RPE cells. The study also elucidated a dose-dependent relationship between the quantity of injected cells and the extent of pigmentation observed.

In terms of visual acuity, electroretinography testing, and microperimetry sensitivity, there was no significant change detected across the patient cohort. This lack of notable improvement or decline in visual functions was hypothesized to be associated with the advanced stage of the disease present in all study participants.

In the field of regenerative medicine, significant research has been directed toward evaluating the safety and tolerability of various scaffolds for human embryonic stem cell-derived retinal pigment epithelium cells. One pioneering approach involved the transplantation of hESC-RPE cells cultured on a synthetic parylene substrate, known as CPCB-RPE1, designed to emulate the characteristics of Bruch’s membrane (NCT02590692) [53]. This novel intervention did not raise any safety concerns in early assessments.

In the majority of subjects (four out of five) receiving this transplant, OCT images revealed alterations indicative of integration between the hESC-RPE cells and the host’s photoreceptors. Notably, none of the eyes that underwent this procedure exhibited a progression in vision loss. Furthermore, one eye recorded a significant improvement, manifesting as a 17-letter increase in visual acuity, while two other eyes showed enhanced fixation capabilities. The researchers posited that the observed structural and functional enhancements might indicate that CPCB-RPE1 has the potential to ameliorate visual function, at least in the short term. This potential benefit was particularly noted in patients suffering from severe vision impairment due to advanced dry AMD [53]. After one year of follow-up, the implant was proven to be safe and well-tolerated by participants with advanced dry AMD [54].

Furthermore, in a more recent longitudinal study of five years, Li et al. [55] thoroughly investigated the long-term safety and tolerability of subretinal transplantation using human embryonic stem cell-derived retinal pigment epithelium in patients diagnosed with Stargardt’s macular dystrophy. Their findings provided substantial evidence supporting the sustained safety and tolerability of this innovative therapeutic approach in treating SMD over an extended period of time [55].

Human embryonic stem cell-derived retinal pigment epithelium cells have been demonstrated to be viable for plating onto a vitronectin-coated polyester membrane, a technique facilitating their transplantation into the subretinal space. In an initial assessment of this method [56], researchers observed its efficacy and safety when applied to two patients with severe wet age-related macular degeneration, monitored over a 12-month period (NCT01691261). The immunosuppressive regime in this case involved the use of fluocinolone. The study noted improvements in both patients in terms of best corrected visual acuity, microperimetry, and reading speed. However, alongside these positive outcomes, the procedure was not without significant complications. These included exposure to the fluocinolone suture, instances of retinal detachment, and an exacerbation of diabetes, attributed to the use of systemic corticoids [56]. This juxtaposition of favorable visual outcomes and serious adverse events highlights the complex balance between therapeutic benefits and potential risks in advanced ocular interventions.

### 4.2. Clinical Trials Using hiPSCs

Following promising outcomes in preclinical studies, human clinical trials were strategically designed and initiated. In 2014, the RIKEN research institute in Japan embarked on a pioneering human clinical trial employing autologous induced pluripotent stem cells to treat a patient with neovascular age-related macular degeneration [57]. The unique aspect of this trial was the autologous nature of the cell transplantation, which eliminated the need for a scaffold and systemic immunosuppression. However, the trial faced an interruption due to the implementation of a new regulatory framework for regenerative medicine in Japan in 2014, despite the patient not experiencing any serious adverse effects [57]. Over a year of follow-up, the patient’s visual acuity remained stable, with no noted improvement. Concerns regarding tumor formation were not realized during the trial. The transplantation of a second patient was not pursued due to the detection of genetic discrepancies, specifically single-nucleotide variations and copy number variants in the hiPSCs, which were absent in the patient’s original somatic cells [30,57].

Subsequently, Mandai et al. [58] (UMIN 000011929) demonstrated the feasibility of transplanting a sheet of autologous RPE cells derived from hiPSCs, sourced from skin fibroblasts, into a patient with wet AMD. Over a 25-month observation period, no adverse events were reported, nor was there any improvement in visual acuity [58].

In a distinct clinical trial, conducted by Sugita et al. [59] (UMIN 000026003), five patients diagnosed with neovascular age-related macular degeneration were enrolled. Induced pluripotent stem cells utilized in this study were derived from a donor with a homozygous human leukocyte antigen (HLA) match. Following pars plana vitrectomy, a suspension of iPSC-derived retinal pigment epithelium cells was administered subretinally. The immunosuppressive regimen was limited to the administration of sub-tenon’s triamcinolone. Over a 52-week monitoring period, no adverse events were reported among the patients. However, all participants developed epiretinal membranes; upon examination, these membranes were found to contain pigmented cells positive for RPE markers. Increased subretinal pigmentation was observed in all subjects, yet in most cases, the pigment deposition was not predominantly located in the macula, likely due to a less than ideal injection location or technique. Sugita and colleagues further acknowledged the complication of graft cells backflowing into the vitreous, highlighting this as an area requiring additional investigation [59].

### 4.3. Clinical Trials Using MSCs

A distinct strategy in stem cell therapy involves the facilitation of functional recovery in the retina’s compromised cells via the introduction of stem cells that exert a paracrine trophic effect. This method, achievable through the employment of mesenchymal stem cells, is not confined to a particular disease, thereby offering a wide range of clinical applications [6].

The encouraging outcomes from experimental studies have paved the way for the initiation of clinical trials. In a prospective phase I study [60], a singular dose of intravitreally administered autologous bone-marrow-derived mesenchymal stem cells was given to three patients with retinitis pigmentosa and two with cone–rod dystrophy. Over a follow-up period of ten months, no significant structural or functional toxic effects were observed in the retina. In this research, conducted by Siqueira et al. [60], four patients with an advanced stage of the disease demonstrated an improvement of one row in best corrected visual acuity one-week post-injection, a benefit that persisted throughout the follow-up period [60].

In a subsequent extension of this study, intravitreal MSCs were administered to 20 patients, who were then monitored for a year. The researchers noted a statistically significant elevation in the patient’s vision-related quality of life scores at the three-month mark. However, by the 12-month evaluation, these scores had reverted to their initial levels, suggesting that the observed improvements were transient [61].

In a separate investigation conducted by Park et al. (NCT01736059) [62], a total of 3.4 million bone-marrow-derived mesenchymal stem cells were intravitreally administered into six eyes suffering from irreversible vision loss due to various conditions, including retinal vascular diseases, hereditary or non-exudative age-related macular degeneration, and retinitis pigmentosa. This therapeutic approach was found to be well-tolerated, with no incidents of intraocular inflammation or proliferation observed. Additionally, there were no declines in electroretinography and best corrected visual acuity results over a six-month follow-up period [62].

With the increasing utilization of mesenchymal stem cells in treatments, there has been a concurrent rise in reported ocular complications associated with this therapy, such as elevated intraocular pressure, hemorrhagic retinopathy, and vitreous hemorrhage [63]. In one particular study [64], the application of autologous bone-marrow-derived MSCs resulted in enhanced visual acuity in two out of three patients with advanced retinitis pigmentosa. However, complications arose in the third patient from the second week post-treatment, including the development of preretinal and vitreal fibrous tissue, shallowing of the anterior chamber, and the formation of a cyclitic membrane, leading to ocular hypotonia. This patient experienced total tractional retinal detachment and a consequent complete loss of vision within three months [64].

In contrast, the suprachoroidal approach proposed by Limoli et al. [65] might mitigate the vitreoretinal complications observed in intravitreal and subretinal MSC applications. In their study, no complications were reported, and visual function was improved in 36 eyes of 25 patients with dry age-related macular degeneration. This was achieved six months after MSCs were administered under a deep scleral flap into the suprachoroidal space, highlighting the potential benefits and reduced risks of this technique [65].

Beyond the direct application of MSCs, recent advancements have highlighted the potential of MSC-derived exosomes and vesicles in retinal therapies [66,67]. MSC-exosomes, which are extracellular vesicles released by MSCs, encapsulate a variety of bioactive molecules including proteins, mRNAs, and microRNAs that can modulate inflammation, angiogenesis, and cellular repair processes [68]. These vesicles harness the paracrine effects of MSCs, potentially offering a cell-free option for treating retinal diseases. For instance, studies have shown that MSC-derived exosomes can promote neuroprotection and angiogenic responses in degenerative conditions of the retina, enhancing retinal cell survival under stress conditions [69] (Table 2). This approach could mitigate some risks associated with direct stem cell transplantation, such as cell proliferation and immune rejection, while still delivering therapeutic benefits.

## 5. Stem Cell Administration Method

Contemporary approaches for stem cell delivery to the ocular region encompass intravitreal, subretinal, and suprachoroidal injections, each with distinct advantages and challenges. Intravitreal injection, a prevalent and relatively straightforward procedure, is extensively utilized for treating retinal diseases, such as exudative AMD. Nevertheless, the integrity of the blood–retinal barrier poses limitations on the effective transport of transplanted stem cells and their secreted neurotrophic factors [71]. Additionally, there is a risk of the therapeutic agents diffusing to non-target areas, potentially inducing fibrous tissue proliferation, retinal detachment, and epiretinal membrane formation [72]. Despite some clinical studies affirming the general safety of stem cell therapy for retinitis pigmentosa patients via this method, it necessitates meticulous consideration prior to application [44].

Conversely, subretinal injection targets the potential space between the retinal pigment epithelium and photoreceptors, providing a more direct approach to the retina. This method, however, entails a pars plana vitrectomy, introducing the risks of RD and associated complications. The successful application of human embryonic stem cell-derived RPE in the subretinal space attests to its relative safety when executed with precision [73]. The delivery of cell suspensions and cells adhered to scaffolds represent two distinct techniques within subretinal injections. While the former is less invasive, the latter, despite necessitating a larger retinotomy for cell delivery, may be secured in place using intraoperative devices, mitigating the risk of postoperative complications such as cell migration, trans-differentiation, and uncontrolled proliferation [74].

The suprachoroidal space (SCS) introduces a novel and less-invasive administration route, accurately targeting the choroid, RPE, and neuroretina, and ensuring high bioavailability [75]. Limoli et al. pioneered the suprachoroidal implantation of stem cells, highlighting its safety profile with no reported ocular adverse events in contrast to the other methods. Furthermore, the SCS facilitates the sustained release of stem cell-derived growth factors, promoting constant secretion to the choroid and retina, which is advantageous for patients requiring multiple cell suspension injections [76,77].

Overall, while each method presents its own set of advantages and potential risks, careful consideration and precision in execution are paramount to optimizing therapeutic outcomes and minimizing complications in stem-cell-based interventions for retinal diseases.

## 6. Cell Suspension or Reconstructed Tissue

The design of a treatment plan remains a critical factor, particularly when dealing with pathologies that include alterations to the Bruch’s membrane. Initially, early methodologies relied on the use of cell suspension [10]. This approach involves delivering stem cells directly into the affected retinal area in a fluid medium, allowing for a diffuse distribution across the retina. The primary mechanism of action for stem cells administered in this manner hinges on their ability to secrete paracrine factors that can modulate the local environment, fostering repair and regeneration. These factors include a range of cytokines and growth factors that promote cell survival, reduce inflammation, and stimulate the resident retinal cells towards repair processes [10,78]. However, science in this field is currently moving towards the use of more sophisticated tissue formation techniques. In order to facilitate the transfer of a preformed epithelium, it is necessary to use a supporting matrix that enables the safe removal of the sheet from the culture plate and subsequent loading into the transplantation device, and to maintain the integrity of the polarized RPE monolayer [13]. This technique, known as the scaffold-based approach, involves attaching stem-cell-derived retinal pigment epithelium cells to a biocompatible scaffold, which not only serves as a structural support but also promotes cell adhesion, proliferation, and differentiation in a controlled manner. Therefore, several characteristics are necessary to facilitate the functioning and viability of human pluripotent stem cell-derived retinal pigment epithelium: thickness, mechanical features such as flexibility and ease of manipulation, permeability, and potential for biodegradation [79]. Various types of scaffolds have been used for different purposes, including the utilization of synthetic polymers, biological materials like Descemet’s membrane or human amniotic membranes, or even the absence of support altogether. In the latter case, retinal pigment epithelial (RPE) cells are permitted to form an epithelium on a collagen covering, which is then broken down to release the sheet [80].

Furthermore, scaffolds have the potential to not only serve as a mechanical support system for transplanted cells but also provide trophic support via the inclusion of substances that enhance cell survival and development. Notwithstanding these benefits, other factors to be taken into account for the effective utilization of scaffolds in the subretinal region include the need for a slender layer measuring between 5 and 90 microns, as well as the possibility of an inflammatory reaction to the implanted substance [81].

Table 3 encapsulates a summary of the materials utilized as scaffolds for retinal pigment epithelium cells. Several research groups are exploring a variety of materials for this purpose, including parylene and various forms of polyester, such as polyethylene terephthalate, lactic-co-glycolic acid, polycaprolactone, poly-L-lactic acid, and vitronectin-coated polyester membranes, to fabricate scaffolds for RPE sheets [56,82]. Concurrently, there is an ongoing evaluation of temporary, biodegradable scaffolds. These are intended to aid in the attachment of RPE cells to the native Bruch’s membrane while concurrently minimizing sustained inflammation [83,84]. In addition, other research factions investigating the use of different substrates, such as amniotic membrane [85] and lenticules derived from femtosecond laser intrastromal lenticule extraction [86]. There is also interest in the creation of scaffold-free sheets using innovative materials like peptide-modified alginate hydrogels [87,88].

Increasingly, cell-derived vesicles, particularly mesenchymal stem cell-derived exosomes [66], are being recognized for their potential in retinal therapies due to their ability to encapsulate and deliver a range of therapeutic molecules. These vesicles are nanosized extracellular vesicles that transport proteins, lipids, and nucleic acids, capable of influencing cell behavior and tissue repair without the complexities and risks associated with whole-cell therapies [66,89]. MSC-derived exosomes have been shown to play a significant role in modulating inflammation, preventing apoptosis, and enhancing angiogenesis, which are crucial for the repair of damaged retinal tissues. Scientific investigations have demonstrated that when these exosomes are introduced into the retinal environment, either independently or in conjunction with scaffold-based systems, they can significantly enhance the therapeutic efficacy by providing localized delivery of growth factors and cytokines directly to the damaged cells [89]. This cell-free approach not only mitigates the risks of cell transplantation, such as immune rejection and tumor formation, but also offers a controlled and sustained release of bioactive factors, potentially improving the integration and functionality of transplanted cells or supporting intrinsic repair mechanisms. The incorporation of MSC-derived vesicles into scaffold systems could offer a dual mechanism of action: structural support from the scaffold and bioactive molecular delivery through the vesicles, thereby enhancing the regenerative capacity of the treatment strategy [89,90].

## 7. Ethical and Safety Issues of Stem-Cell-Based Therapy

The exploration of stem-cell-based therapies for retinal regeneration, particularly involving human embryonic stem cells and induced pluripotent stem cells, is surrounded by complex ethical and safety considerations. The use of hESCs has historically sparked considerable ethical debate due to their derivation methods, which require the destruction of human embryos. This issue has led to significant ethical concerns and diverse political and policy responses across different countries [91,92,93]. In some regions, this has led to strict regulations or outright bans on hESC research, while in others, it has been politically charged, used in broader debates over human rights and medical ethics. Furthermore, the advent of iPSC technology, which involves reprogramming adult cells to an embryonic-like state, was initially perceived as a less ethically fraught alternative. However, iPSCs also harbor the potential for unlimited differentiation and could theoretically be used for human cloning, raising new ethical dilemmas concerning identity and the potential creation of human embryos for research purposes [92].

A significant safety issue with iPSC transplantation and the use of iPSC-derived cells is the possibility of unintended differentiation and malignant transformation. To address this, there is a crucial need to refine and optimize protocols for iPSC differentiation. Ensuring the purity of iPSC-derived differentiated cell populations is essential before they can be deemed safe for clinical application. This is vital to prevent any oncogenic potential these cells might harbor, which could have detrimental effects on patients [93].

Mesenchymal stem cells have also emerged as a popular choice in stem cell therapy, often touted as a universal remedy in various medical treatments across the globe. However, their safety profile is not fully understood, particularly concerning their potential to promote tumor growth and metastasis. Therefore, research employing MSCs must prioritize continuous monitoring and extensive long-term follow-up, especially in animal models. This vigilance is necessary to uncover any pro-tumorigenic or other ad-verse effects of MSC-based therapy. Such comprehensive evaluations are essential to establish a robust understanding of the implications of stem cell therapies and to ensure that their therapeutic benefits do not come at the cost of patient safety [60,93].

While the field of stem cell therapy for retinal regeneration is burgeoning with potential, it navigates a complex landscape of ethical dilemmas and safety challenges. Another ethical and safety concern is raised by the use of stem cells in treating degenerative retinal diseases in clinics not officially approved for such therapies. While the potential of stem cell therapy offers hope for conditions with limited treatment options, administering these treatments outside of approved clinical trials or recognized hospitals can jeopardize patient safety. Without rigorous regulatory oversight, patients may be exposed to unproven interventions lacking evidence of efficacy or safety, risking possible adverse effects without the guarantee of therapeutic benefit [94]. It underscores the necessity of adhering to established clinical guidelines and regulatory approvals, ensuring that stem cell therapies are both safe and effective before applying them in a broader clinical setting. Addressing these concerns requires a balanced approach, ensuring that these innovative therapies are both ethically sound and safe for clinical use [94].

## 8. Conclusions

Over the last two to three decades, a substantial body of research has convincingly shown that transplantation of the retinal pigment epithelium (RPE) can at least partially restore retinal structure, function, and subjective visual perception in individuals with retinal degenerative diseases. Breakthroughs in foundational sciences and translational research domains, including stem cell biology, retinal surgery, non-invasive retinal imaging, retinal physiology, and vision science, have brought the field to the brink of human clinical trials capable of offering therapies to restore vision. A number of early-phase human clinical trials are currently underway globally, and their outcomes are anticipated to be groundbreaking. However, ongoing research and collaboration among funding bodies, academic institutions, and industry partners are imperative to ensure successful outcomes.

Primary concerns in human clinical trials related to RPE cell layer transplantation involve several key aspects: ensuring the longevity of the donor RPE in the host to justify the risks associated with implantation and cell-based therapy; maintaining the polarity and functional integrity of the donor RPE akin to normal RPE cells; preventing further degeneration of the donor RPE cells that may be associated with the disease process; and determining the most effective technique for delivering the RPE cells into the subretinal space. It is anticipated that the numerous concerns presently raised will find their resolutions in the forthcoming period.

Moreover, as the field advances, the importance of rigorous patient selection, ethical considerations, and long-term follow-up cannot be overstated. Technological innovations and improvements in surgical techniques promise to refine the delivery and integration of RPE cells, potentially enhancing treatment outcomes. Concurrently, ethical practices and thorough patient education will remain paramount to navigate the complex landscape of stem cell therapy with transparency and integrity. Ultimately, the success of these pioneering therapies will depend on sustained collaborative efforts, ensuring that the vision of restoring sight through stem cell therapy becomes a safe and accessible reality for those in need.

## Figures and Tables

**Table 1:** Summary table of the current human clinical trials.

Number	Disease	Cell Type	Phase	No. of Patients	Administration Method	Status
NCT03305029	AMD (GA)	hESC-RPE (SCNT-hES-RPE)	I	3	N/A	Unknown
NCT02903576	AMD; SMD	hESC-RPE	I/II	15	suspension vs. scaffold	Completed
NCT03046407	AMD (GA)	hESC-RPE	I	10	N/A	Unknown
NCT02755428	AMD	hESC-RPE (MA09-hRPE)	I	10	N/A	Unknown
NCT02286089	AMD (GA)	hESC-RPE	I/IIa	24	suspension	Ongoing
NCT01344993	AMD	hESC-RPE (MA09-hRPE)	I/II	13	suspension	Completed
NCT02463344	AMD	hESC-RPE	I/II	11	subretinal injection	Completed
NCT01674829	AMD	hESC-RPE	I/IIa	10	N/A	Completed
NCT01691261	AMD	hESC-RPE	I	10	N/A	Recruiting
NCT03102138	AMD	hESC-RPE	Obs.	10	N/A	Ongoing
NCT02590692	AMD (GA)	hESC-RPE	I/IIa	16	scaffold (parylene membrane)	Unknown
NCT02749734	AMD; SMD	hESC-RPE	I/II	15	N/A	Unknown
NCT01345006	SMD	hESC-RPE	I/II	13	subretinal injection	Completed
NCT01469832	SMD	hESC-RPE	I/II	12	N/A	Completed
NCT01625559	SMD	hESC-RPE	I	3	N/A	Completed
NCT02941991	SMD	hESC-RPE	Obs.	12	subretinal injection	Completed
NCT02445612	SMD	hESC-RPE	Obs.	13	subretinal injection	Completed
NCT03944239	RP	hESC-RPE	I	10	N/A	Unknown
NCT03963154	RP	hESC-RPE	I/II	7	N/A	Ongoing
NCT05991986	AMD	iPSC	Obs.	10	N/A	Ongoing
NCT04339764	AMD (GA)	iPSC-RPE	I/II	20	scaffold	Recruiting
NCT02464956	AMD	iPSC-RPE	Obs.	3	N/A	Completed
NCT05445063	AMD (GA)	iPSC-RPE	I	10	N/A	Recruiting
NCT03372746	AMD	iPSC	Obs.	187	N/A	Completed
NCT02016508	AMD	hBM-MSC	I/II	1	intravitreal injection	Unknown
NCT05712148	RP	MSC	I/II	15	suprachoroidal implantation	Completed
NCT05786287	RP	UC-MSC	Obs.	18	N/A	Ongoing
NCT04315025	RP	UC-MSC	I/II	18	suspension (peribulbar injection)	Completed
NCT01531348	RP	hBM-MSC	I	14	subretinal injection	Completed
NCT04763369	RP	UC-MSC	II	50	sub-tenon space injection	Unknown
NCT01736059	RP; AMD	BM-CD34+	I	15	intravitreal injection	Ongoing

Abbreviation: AMD, age-related macular degeneration; GA, geographic atrophy; SMD, Stargardt’s macular dystrophy; RP, retinitis pigmentosa; hESC, human embryonic stem cell; iPSC, induced pluripotent stem cell; MSC, mesenchymal stem cell; RPE, retinal pigment epithelium; hBM, human bone marrow; UC, umbilical cord; Obs, Observational; N/A, Not Applicable.

**Table 2:** Summary of published clinical study results on stem cell therapies.

Disease	Stem Cell Type	Clinical Trials and Phases	Key Findings and Outcomes	Challenges and Considerations
Age-Related Macular Degeneration (AMD)	ESCs, iPSCs, MSCs	NCT01345006 (Phase I/II), NCT01344993 (Phase I/II), [49,50] NCT01691261 (Phase I) [56]	Trials indicate safety and efficacy of ESCs and iPSCs in replacing damaged RPE cells. Some improvements in visual acuity noted.	Managing immune rejection, ensuring integration and long-term safety, ethical concerns with ESCs.
Retinitis Pigmentosa	ESCs, iPSCs, MSCs	NCT01531348 (Phase I) [70], NCT01736059 (Phase I) [62]	Stem cell therapies shown to slow disease progression with potential restoration of some visual functions. MSCs highlighted for their neuroprotective effects.	Genetic stability of iPSCs, ethical considerations, technical delivery challenges.
Stargardt’s Disease	ESCs, iPSCs, MSCs	NCT01345006 (Phase I/II) [49,50], NCT01469832 (Phase I/II) [52]	iPSC trials show potential in restoring visual function. Positive safety profiles and functional improvements in early results	Addressing immune rejection, long-term viability of transplanted cells, ethical and technical challenges.

**Table 3:** Materials used as scaffolds for RPE sheets.

Types of Materials
Parylene [82]
Polyethylene terephthalate [79]
Lactic-co-glycolic acid [79]
Polycaprolactone [79]
Poly-L-lactic acid [79]
Vitronectin-coated polyester membrane [56]
Amniotic membrane [85]
Femtosecond-derived lenticule [86]

## Data Availability

Not applicable.
